# Early‐stage 2K1C renovascular hypertension does not increase global MHC‐II or F4/80 abundance in the non‐clipped kidney but reveals focal F4/80^+^ induction

**DOI:** 10.1113/EP093957

**Published:** 2026-08-29

**Authors:** Pilar Cárdenas, Allison C. León, Juan Castillo‐Geraldo, Catalina Cid‐Salinas, Javiera Saez, Stefanny Figueroa, Javier Reyes, Emma Rosenkoetter, Suarybell Guzmán Román, Carmen De Miguel, Cristián A. Amador, Alexis A Gonzalez

**Affiliations:** ^1^ Instituto de Química Pontificia Universidad Católica de Valparaíso Valparaíso Chile; ^2^ Facultad de Ciencias Universidad San Sebastián Santiago Chile; ^3^ Section of Cardio‐Renal Physiology and Medicine, Division of Nephrology, Department of Medicine, Heersink School of Medicine University of Alabama at Birmingham Birmingham Alabama USA; ^4^ Centro de Investigación Interdisciplinaria en Biomedicina, Biotecnología y Bienestar (CID3B) Pontificia Universidad Católica de Valparaíso Valparaíso Chile

**Keywords:** 2K1C, antigen‐presenting cells, dendritic cells, F4/80, intrarenal RAS, macrophages, MHC‐II, renovascular hypertension

## Abstract

The two‐kidney, one‐clip (2K1C) model produces renovascular hypertension. The effects on the clipped kidney (CK) are well described, while the non‐clipped kidney (NCK) is used as a model to evaluate the effects of high blood pressure. Although most of these effects have been described at later stages of 2K1C hypertension, it remains unclear whether immune antigen‐presenting cells (APCs) expand early in the NCK during the first stages of 2K1C hypertension. Our objective was to determine whether the 2K1C model increases the presence and/or distribution of APCs in the NCK during the early phase of renovascular hypertension. Male C57BL/6J mice underwent sham surgery or 2K1C surgery (*n* = 6). Blood pressure was assessed, and NCKs were collected on day 7. Global expression of major histocompatibility complex (MHC)‐II (APC marker) and F4/80 (monocyte/macrophage lineage) was quantified by immunoblotting, while spatial distribution was evaluated by immunohistochemistry (IHC) and morphological analysis. 2K1C produced a significant increase in blood pressure in the NCK without albuminuria. Immunoblot experiments showed no significant differences in global MHC‐II or F4/80 levels between sham and 2K1C groups in the NCK. However, IHC revealed a focal increase in F4/80^+^ signal and a significant rise in F4/80^+^ area fraction, consistent with localized monocytic/macrophage enrichment not captured by whole‐tissue homogenates from NCK. By contrast, F4/80 immunostaining was observed in conspicuous resident cells. After 7 days of 2K1C hypertension, contralateral renal APC expansion is not evident at the global protein level, yet focal F4/80^+^ enrichment suggests early regional and focal immune remodelling that may precede later overt inflammation.

## Abstract (highlights)

- **What is the central question of this study?**
  Does the early phase of two‐kidney, one‐clip (2K1C) renovascular hypertension cause expansion or redistribution of antigen‐presenting cells (APCs) in the non‐clipped kidney before overt renal injury?
- **What is the main finding and its importance?**
  Early 2K1C hypertension did not increase overall renal MHC‐II or F4/80 expression but produced focal F4/80^+^ macrophage enrichment in the non‐clipped kidney, identifying localized immune remodelling as an early event preceding overt inflammation and suggesting therapeutic targets during hypertension initiation.

## 1 INTRODUCTION

Hypertension remains a leading modifiable risk factor for cardiovascular and renal morbidity worldwide (Dominiczak & Delles, 2025). Beyond classical haemodynamic mechanisms, cumulative evidence supports a central contribution of immune and inflammatory pathways to the initiation and maintenance of elevated blood pressure (Le et al., 1999; Li et al., 2005; Solak et al., 2016; Wang et al., 2024). Both innate and adaptive immune cells participate in vascular dysfunction, renal injury and altered sodium handling, thereby influencing long‐term arterial pressure regulation (Chambrey et al., 2013; Mattson et al., 2021). The kidney occupies a pivotal position in this paradigm, serving not only as a target of hypertensive damage but also as a critical determinant of blood pressure homeostasis by regulating extracellular volume and electrolyte balance (De Miguel et al., 2010; Ertuglu & Kirabo, 2022; Makhanova et al., 2010; Wang et al., 2024).

Among experimental models, the two‐kidney, one‐clip (2K1C) renovascular model has been extensively used to dissect the interplay between renal perfusion, renin–angiotensin system (RAS) activation and hypertension (Wang et al., 1999). Partial stenosis of one renal artery leads to hypoperfusion of the clipped kidney, triggering robust renin release and sustained elevation of circulating angiotensin II (Ang II) (Navar & Harrison‐Bernard, 2000; Navar et al., 1999). This endocrine activation is accompanied by strong stimulation of the intrarenal RAS, which operates in a paracrine and autocrine manner, thereby achieving Ang II concentrations substantially higher than those found in the circulation (Prieto et al., 2003; Ryuzaki et al., 2011). Consequently, Ang II acts not only as a vasoconstrictor hormone but also as a pleiotropic signalling molecule that influences oxidative stress, fibrosis and immune cell behaviour (Ponchon & Elghozi, 1996). Ang II also promotes inflammatory responses primarily through AT1 receptor signalling, activating transcriptional mechanisms leading to increases in nuclear factor‐κB, activator protein‐1 and signal transducer and activator of transcription pathways and inducing expression of cytokines and chemokines including tumour necrosis factor‐α, interleukin (IL)‐6, monocyte chemoattractant protein‐1 and transforming growth factor‐β (Mezzano et al., 2001; Ruiz‐Ortega, Lorenzo, Rupérez et al., 2001, Ruiz‐Ortega, Lorenzo, Suzuki 2001). These mediators establish a microenvironment that favours recruitment, activation and functional polarization of immune cells within the kidney (Abbas et al., 2025; Adeshara et al., 2025; Arendshorst et al., 2024; Bunchman et al., 1991; Burns‐Ray et al., 2025; Cardenas et al., 2025; Chen et al., 2022, 2025; de Winter et al., 2026; Rodriguez‐Iturbe et al., 2002). In experimental hypertension, immune cell accumulation correlates with renal structural damage, albuminuria and progressive decline in renal function, supporting a mechanistic link between immune activation and hypertensive renal injury (Crowley et al., 2006; Rodriguez‐Iturbe et al., 2002).

Antigen‐presenting cells (APCs), principally dendritic cells (DCs) and macrophages, are increasingly recognized as central regulators of hypertensive inflammation. Under physiological conditions, the kidney contains a dense network of resident mononuclear phagocytes that perform immune surveillance, clear debris and contribute to tissue homeostasis (Kurts et al., 2020). These cells typically express major histocompatibility complex (MHC) class II molecules, reflecting their capacity to present antigen, while macrophage‐lineage cells are commonly identified by expression of F4/80 in mice (Guan et al., 2025). Importantly, renal APC populations are heterogeneous and exhibit considerable phenotypic plasticity, with overlapping expression of markers traditionally associated with macrophages or DCs (Liu et al., 2001; Qian & Cao, 2018).

In hypertensive conditions, APCs acquire proinflammatory properties and can actively shape disease progression (Peter et al., 2024). Experimental depletion of monocytes/macrophages attenuates Ang II‐induced hypertension, reduces renal cytokine expression and limits tissue injury (Peter et al., 2025). Similarly, manipulation of DC populations alters blood pressure responses and vascular inflammation, underscoring their functional relevance (Araos et al., 2019). More recently, evidence has emerged that renal APCs can directly modulate sodium handling and thereby influence blood pressure regulation (Hevia et al., 2018). Adoptive transfer of renal CD11c^+^ DCs from Ang II‐hypertensive mice has been shown to transmit aspects of the hypertensive phenotype to normotensive recipients, whereas renal macrophages do not exert the same effect, highlighting a specific prohypertensive role for DCs (Hevia et al., 2018).

Although immune involvement in hypertension is now well established, the temporal sequence of immune activation within different renal compartments remains incompletely defined. In the 2K1C model, most studies have focused on intermediate or late stages (≥14 days), when immune‐cell infiltration and renal remodelling are clearly detectable. At these time points, increased numbers of macrophages and DCs are observed predominantly in the clipped kidney, with more modest changes in the contralateral kidney. However, hypertension becomes established within the first week after clip placement, raising the question of whether immune alterations in the contralateral kidney begin earlier than traditionally appreciated.

The contralateral kidney, or non‐clipped kidney (NCK), represents a particular physiological condition during renovascular hypertension. While the clipped kidney experiences hypoperfusion and ischaemic stress with well‐described mechanisms of RAS activation (Fedorova et al., 2013; Mackenzie et al., 1985), the NCK is exposed to elevated systemic blood pressure and circulating Ang II, which in turn activate intrarenal RAS (Prieto et al., 2011; Prieto‐Carrasquero et al., 2008). In addition, the NCK must compensate to preserve overall renal excretory function, serving as a physiological model to study hypertension‐related mechanisms affecting the hyperfiltrating kidney (Mackenzie et al., 1985). Most of these effects related to immune responses have been described over long‐term periods (Haller et al., 1997). These conditions may exhibit early and regionally restricted immune adaptations that precede overt inflammatory infiltration or global increases in immune‐cell abundance. Such early changes might be spatially confined to peritubular or interstitial niches and therefore difficult to detect using whole‐tissue homogenization approaches.

Methodological considerations are particularly relevant in this context. Western blotting provides quantitative information on global protein abundance but lacks spatial resolution and may fail to capture focal microdomain alterations. In contrast, immunohistochemistry enables visualization of regional cellular distribution and identification of localized enrichment of immune populations, even when total protein levels remain unchanged. Combining these approaches offers a strategy to distinguish between widespread immune expansion and subtle, spatially restricted remodelling.

Based on this framework, we hypothesized that early renovascular hypertension induced by the 2K1C model increases the presence and/or distribution of APCs in the NCK during the first week of disease development. To test this hypothesis, we assessed global expression of MHC‐II and F4/80 by western blot and evaluated their spatial localization by immunohistochemistry in NCKs 7 days after clip placement. By defining whether APC‐associated signals emerge at this early stage, this study aims to refine the temporal model of immune involvement in renovascular hypertension and provide insight into the earliest immune adaptations occurring in the NCK.

## 2 METHODS

### 2.1 Ethical approval

All procedures were approved by the Institutional Bioethics Committee of the Pontificia Universidad Católica de Valparaíso (BIOEPUCV‐BA 482–2022) and followed NIH guidelines for the care and use of laboratory animals.

### 2.2 Animals

All animals used in this study were obtained from the breeding colony maintained at the Animal Facility of the Centro Interdisciplinario de Neurociencia de Valparaíso (CINV), Valparaíso, Chile, where they were bred and housed under controlled environmental conditions until the experimental procedures were performed. Male C57BL/6J mice (8–10 weeks old) were housed under controlled conditions (21°C, 50% humidity, 12:12 h light–dark cycle) with free access to standard chow and water. Animals were randomly assigned to two experimental groups: sham surgery controls and 2K1C. An a priori sample size calculation, based on effect sizes obtained from our previous studies using the same 2K1C renovascular hypertension model and comparable physiological and molecular endpoints, indicated that six animals per group (*n* = 6) would provide 80% statistical power to detect biologically relevant differences (α = 0.05). Because the Goldblatt (2K1C) surgical procedure is associated with occasional perioperative mortality or unsuccessful clip placement, approximately two additional animals per group were initially included to compensate for potential losses and ensure that the predetermined sample size was achieved. This approach also adheres to the principles of reduction in animal experimentation.

### 2.3 Urinary albumin

Urinary albumin was measured by an ELISA kit (cat. no. EEL119, Thermo Fisher Scientific, Waltham, MA, USA) according to the manufacturer's instructions. Results are expressed as micrograms per day.

### 2.4 Induction of renovascular hypertension (2K1C)

Mice were anaesthetized with 2% inhaled isoflurane and placed in a lateral position. A flank incision was performed to expose the left kidney via a retroperitoneal approach. The renal artery was carefully dissected and separated from the vein. A silver clip with an internal diameter of 0.13 mm was placed around the artery to induce partial stenosis. The kidney was repositioned, and muscle and skin were sutured. Sham‐operated mice underwent identical surgical manipulation without placement of the clip. Animals were monitored daily for general health, body posture, activity, grooming behaviour, food and water intake, and wound integrity. Postoperative discomfort was assessed using the Mouse Grimace Scale. Analgesia was provided when required.

### 2.5 Blood pressure measurement

On day 7 after surgery, systolic and diastolic blood pressure were measured by tail‐cuff plethysmography (CODA®, Kent Scientific, Torrington, CT, USA). Mice were acclimated to the procedure with a training period, and at least five valid measurements were obtained per animal.

### 2.6 Tissue collection

On day 7, animals were deeply anaesthetized with 2% isoflurane and euthanized by cervical dislocation. Both kidneys were rapidly excised, rinsed in cold saline and sectioned. Portions were snap‐frozen for protein analysis or fixed for histological studies. Analyses presented here focused on the contralateral (right) kidney.

### 2.7 Protein extraction and western blotting

Renal cortex and medulla were homogenized in RIPA buffer supplemented with protease inhibitors. Lysates were centrifuged (8000 *g*, 20 min, 4°C), and supernatants were collected. Protein concentration was determined using a bicinchoninic acid (BCA) assay. Twenty‐five micrograms of protein was separated by SDS–PAGE and transferred to polyvinylidene difluoride membranes. Membranes were blocked and incubated overnight with primary antibodies against MHC‐II (sc‐32247, Santa Cruz Biotechnology, Dallas, TX, USA) at 1:400 dilutions; F4/80 (cat. no. MCA497R, Bio‐Rad Laboratories, UK) at 1:200 dilutions; and β‐actin (sc‐47778, Santa Cruz Biotechnology) at 1:200 dilutions. After incubation with horseradish peroxidase‐conjugated secondary antibodies, signals were detected by enhanced chemiluminescence. Band intensity was quantified by densitometry and normalized to β‐actin.

### 2.8 Histology and immunohistochemistry

Kidney fragments were fixed in Bouin's solution (12–16 h), dehydrated, embedded in paraffin, and sectioned at 5 µm. Sections were stained with Haematoxylin–eosin (H&E) to evaluate general renal morphology. For immunohistochemistry, sections were deparaffinized, rehydrated, subjected to antigen retrieval and incubated with primary antibodies against F4/80 (cat. no. MCA497R, Bio‐Rad) at 1:200 dilution and MHC‐II (sc‐32247, Santa Cruz Biotechnology) at 1:200 dilution. Detection was performed using polymer‐based secondary antibodies and 3,3′‐diaminobenzidine as chromogen. Sections were counterstained with haematoxylin. Images were captured using light microscopy. For F4/80, morphometric analysis of whole kidney scans was performed using cellSens Imaging software (Evident Scientific, Waltham, MA, USA) to quantify the percentage of area stained positive for F4/80 per kidney. ImageJ was used to quantify the renal expression of MHC‐II. Representative sections from cortex and medulla were used.

### 2.9 Statistical analysis

Data are presented as means ± SD. Comparisons between Sham and 2K1C groups were performed using a two‐tailed Mann–Whitney test. *P* < 0.05 was considered statistically significant.

## 3 RESULTS

### 3.1 Tissue analysis at day 7

**FIGURE 1:**
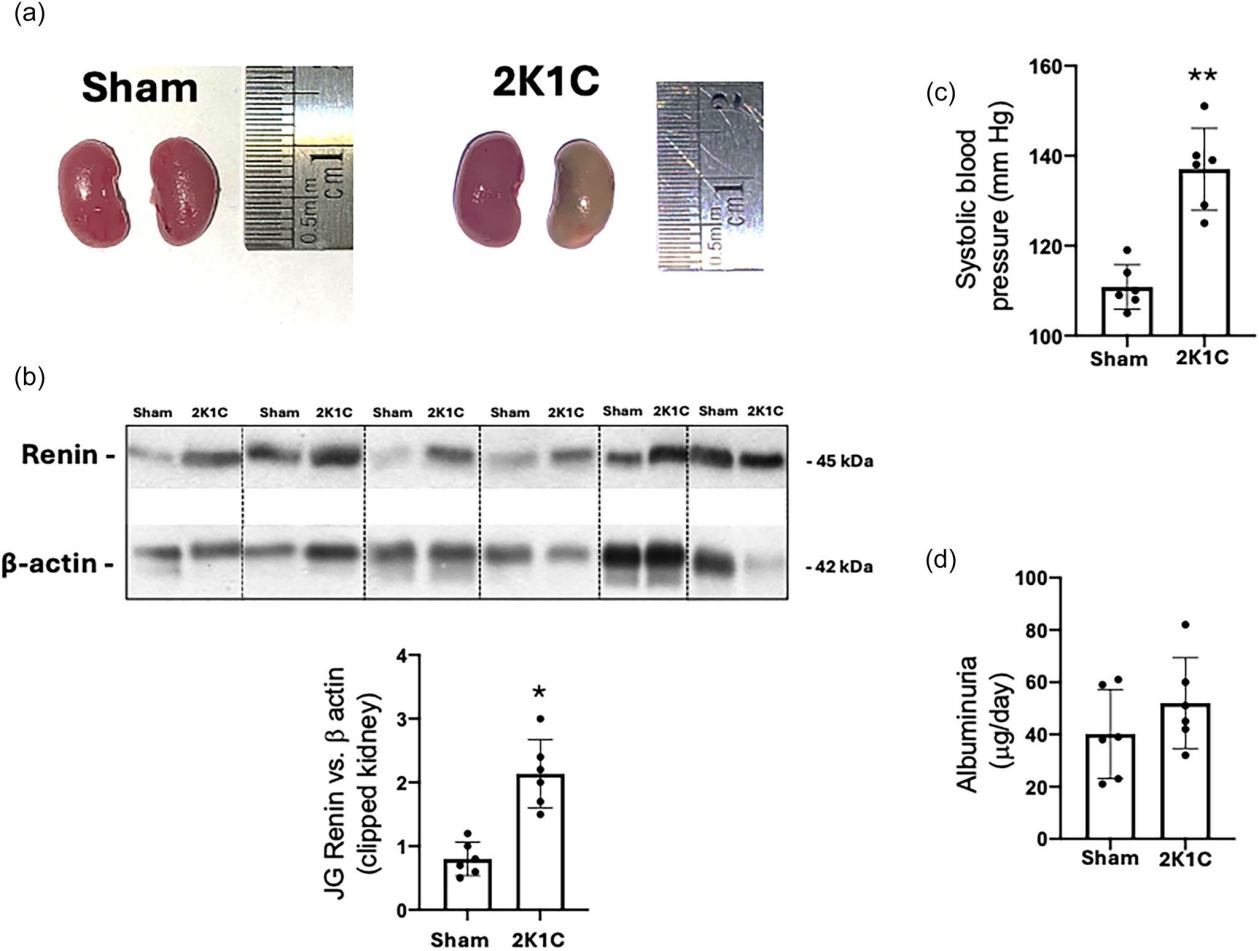
Characterization of the 2K1C model at day 7. (a) Representative images of kidneys from Sham and 2K1C mice showing no differences in kidney size. In contrast, the clipped kidney (right) from 2K1C mice exhibited a marked change in colour, consistent with reduced renal perfusion. (b) Representative western blots (*n* = 6 per group) demonstrate increased renin protein abundance in the clipped kidneys of 2K1C mice compared to controls. Cropped immunoblots corresponding to each animal and their respective β‐actin loading controls are shown, with densitometric quantification presented below. (c) Systolic blood pressure was significantly elevated in 2K1C mice, confirming the hypertensive phenotype. (d) Urinary albumin excretion did not differ significantly between groups. Data are presented as means ± SD. *P < 0.05 and **P < 0.01 versus Sham.

To validate the Goldblatt renovascular model, we analysed kidney appearance in sham‐treated mice and mice with the 2K1C surgery. As shown in Figure 1a, the left kidney from 2K1C mice has an altered colour showing signs of hypoxia, with no reduction in size. Since left renal artery clipping leads to activation of juxtaglomerular (JG) signalling, we evaluated renin abundance in cortical renal tissues from Sham and 2K1C mice by immunoblotting. Renin versus β‐actin blot analysis demonstrated markedly increased cortical renin expression in the clipped kidney of 2K1C animals, indicating activation of the systemic RAS at day 7 (2K1C: 2.12 ± 0.8 vs. Sham 0.84 ± 0.2, *P* = 0.021, Figure 1b). Tail‐cuff plethysmography revealed a significant increase in systolic and diastolic blood pressure in 2K1C mice compared with Sham controls (2K1C: 137 ± 9 vs. Sham 108 ± 6 mmHg, *P* = 0.011, Figure 1c). We further determined the presence of incipient glomerular damage by analysing albuminuria. As shown in Figure 1d, no statistical differences were found at day 7 (2K1C: 52 ± 17 vs. 40 ± 16 µg/day, non‐significant). These findings confirm that the renovascular model was physiologically established, characterized by strong RAS activation in the clipped kidney (increased JG renin) with no albuminuria at the time of analysis.

### 3.2 Histological characteristics of the NCK after 7 days of 2K1C surgery

**FIGURE 2:**
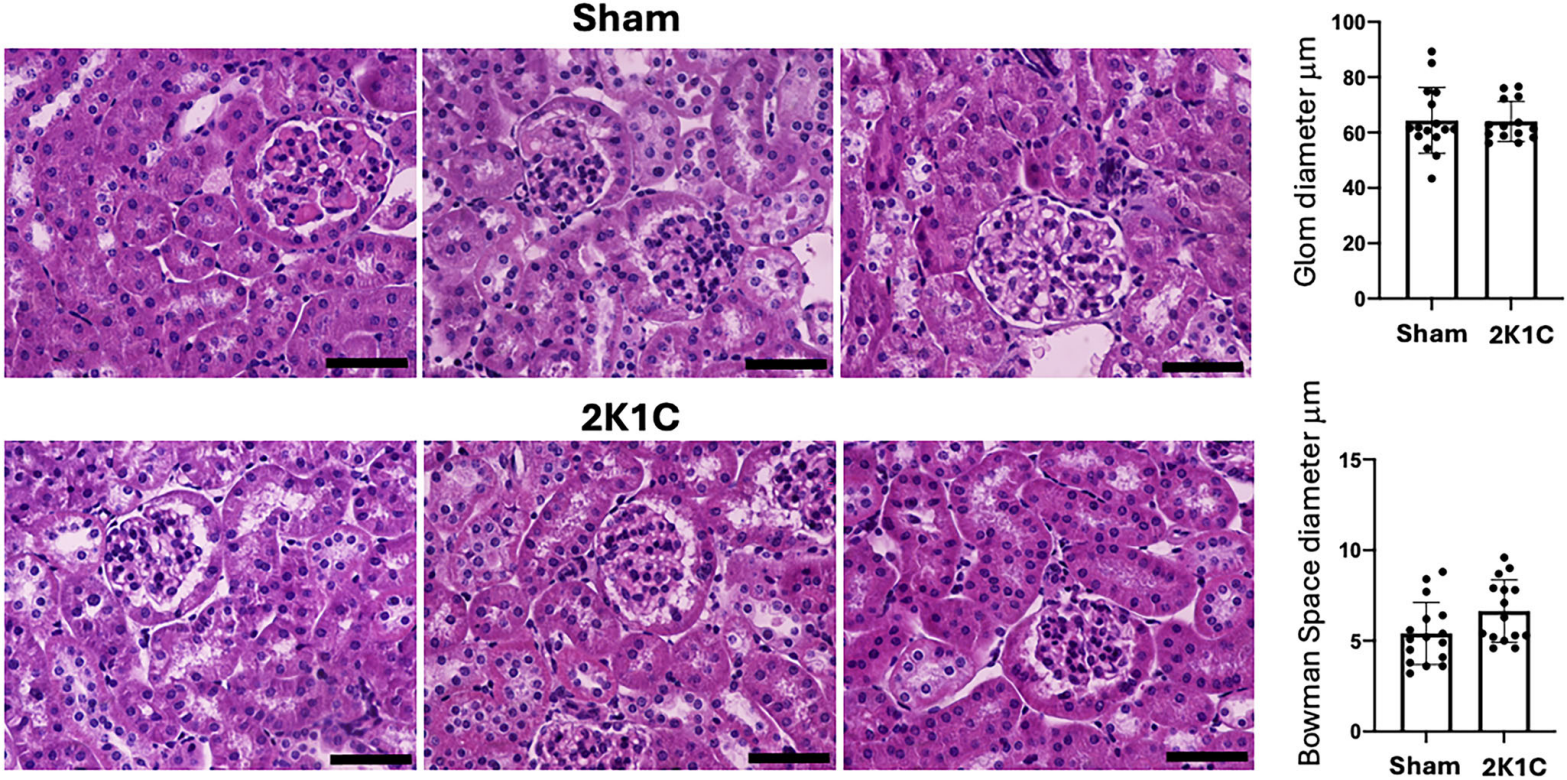
Representative H&E‐stained kidney sections from Sham and 2K1C mice demonstrating preserved renal architecture in both groups. Quantitative morphometric analysis revealed no significant differences in glomerular diameter or Bowman's space diameter between Sham and 2K1C mice, indicating the absence of overt glomerular structural remodelling at this early stage of renovascular hypertension. Data are presented as means ± SD from 2–3 representative fields per animal (*n* = 6 per group). Scale bar: 20 µm.

H&E‐stained sections of NCKs showed preserved architecture in both groups. Glomeruli exhibited normal size and structure, without mesangial expansion or Bowman's space enlargement. No significant differences between groups were found in glomerular diameter (2K1C: 64 ± 12 vs Sham 65 ± 1 µm, *P* = 0,523) and Bowman's space area (2K1C: 6.6 ± 1.6 vs. Sham 5.4 ± 1.7 µm^2^, *P* = 0.312). Figure 2 shows three representative images of each group. Compared to sham‐operated kidneys, the NCKs from 2K1C mice did not show major changes in kidney size or weight (data not shown).

### 3.3 Global expression of F4/80 and MHC‐II in the NCK

**FIGURE 3:**
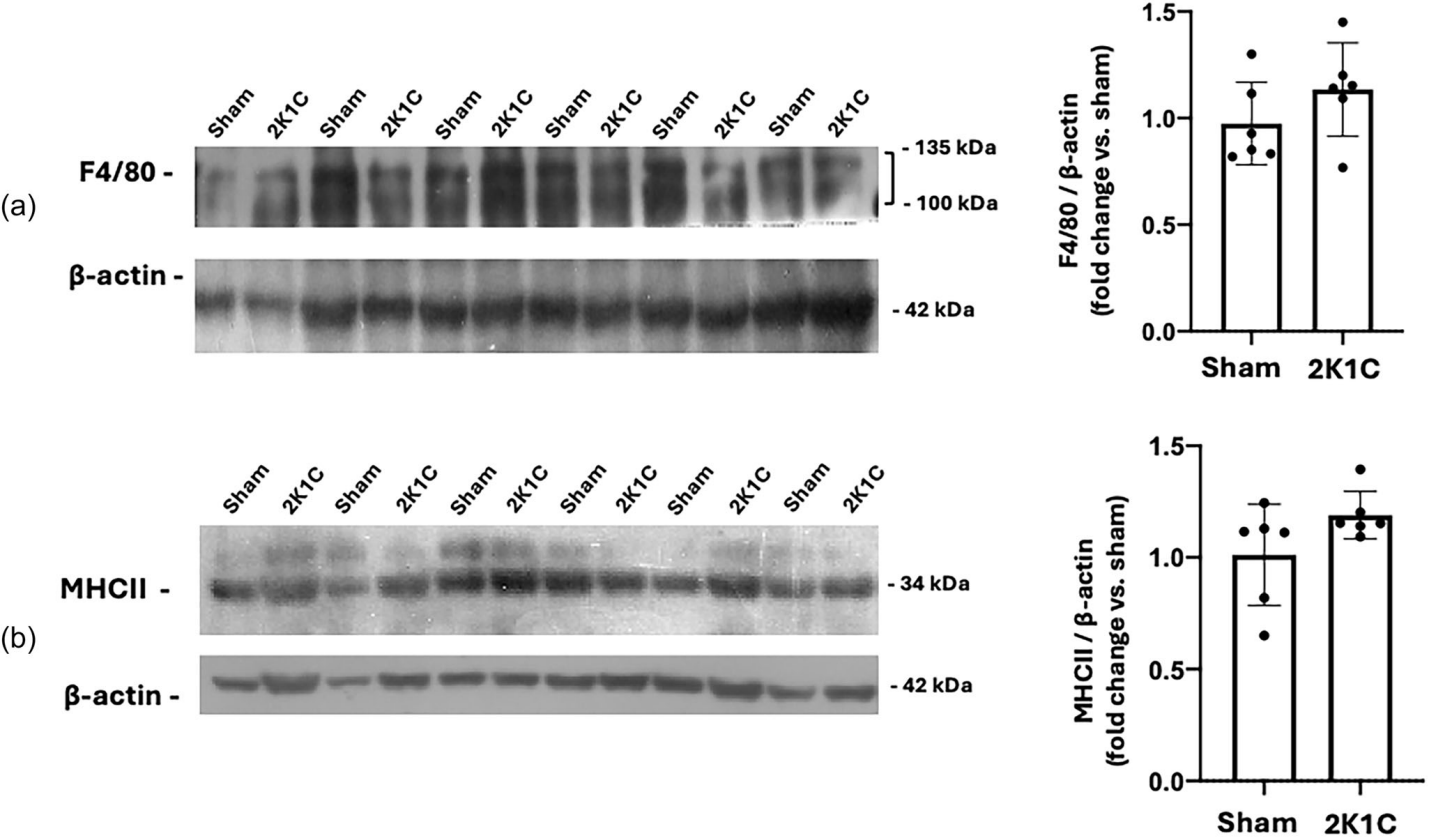
Protein expression of inflammatory markers in kidneys from Sham and 2K1C mice. Representative western blots and densitometric analysis showed no significant differences in renal F4/80 (a) or MHCII (b) protein abundance between Sham and 2K1C groups. Data are presented as fold change versus Sham and expressed as means ± SD (*n* = 6).

Western blot and densitometric quantification of F4/80 (Figure 3a) bands revealed no significant differences in NCKs between sham and 2K1C groups (2K1C: 1.1 ± 0.2 vs. Sham: 1.0 ± 0.1, *P* = 0.081). The same results were observed for MHC‐II (2K1C: 1.2 ± 0.1 vs. Sham: 1.0 ± 0.2, *P* = 0.112, Figure 3b). Interestingly, F4/80 and MHC‐II proteins were readily detectable in NCKs from both groups. Thus, total tissue abundance of macrophage‐lineage marker F4/80 and MHC‐II remains unchanged during the first week of 2K1C hypertension.

### 3.4 Spatial distribution of F4/80^+^ reveals specific focal increases in the NCK of 2K1C mice

**FIGURE 4:**
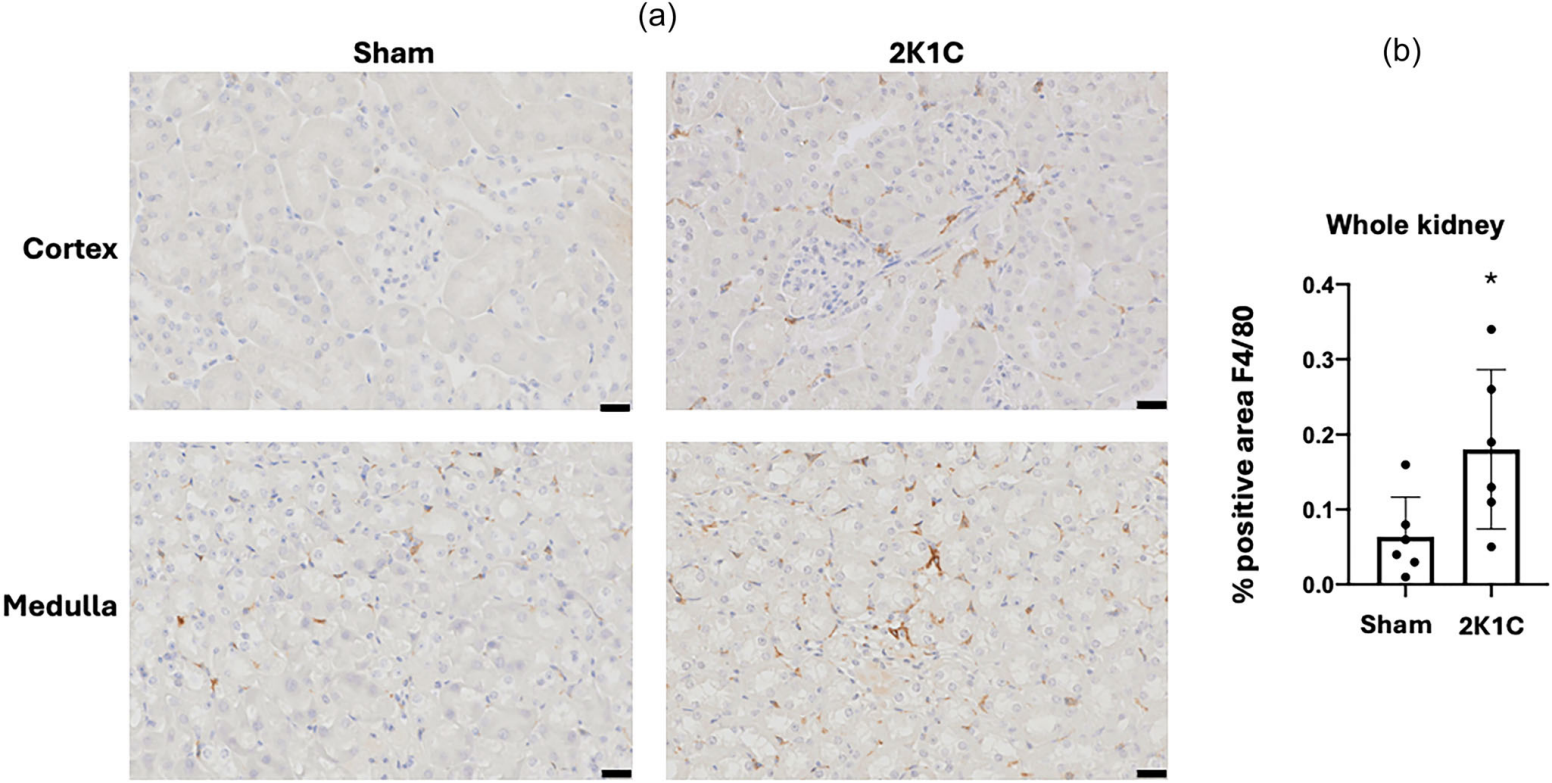
(a) Representative immunohistochemical staining for F4/80 in renal cortex and medulla from Sham (*n* = 7) and 2K1C (*n* = 6) mice. Increased F4/80‐positive staining was observed in kidneys from 2K1C animals, particularly in the medulla, indicating enhanced macrophage infiltration. (b) Quantification of whole‐kidney F4/80‐positive area demonstrated a significant increase (*P* = 0.012) in F4/80^+^ area fraction in 2K1C compared with Sham kidneys. Data are presented as means ± SD. Scale bar: 20 µm. *P < 0.05 versus Sham.

In Sham kidneys, F4/80^+^ cells were sparsely distributed within cortical and medullary interstitium, consistent with resident macrophages. In contrast, NCKs from 2K1C mice displayed visibly increased F4/80 staining in discrete cortical and medullary regions, particularly in interstitial and peritubular areas (Figure 4a). Quantitative morphometry of the whole kidney demonstrated a significant increase in F4/80^+^ area fraction in 2K1C compared with Sham kidneys (2K1C: 0.18 ± 0.04 vs. Sham: 0.05 ± 0.02 %, *P* = 0.012, Figure 4b). These data reveal regional enrichment of macrophage‐lineage cells despite unchanged global F4/80 protein abundance.

### 3.5 Spatial distribution of MHC‐II^+^ cells

**FIGURE 5:**
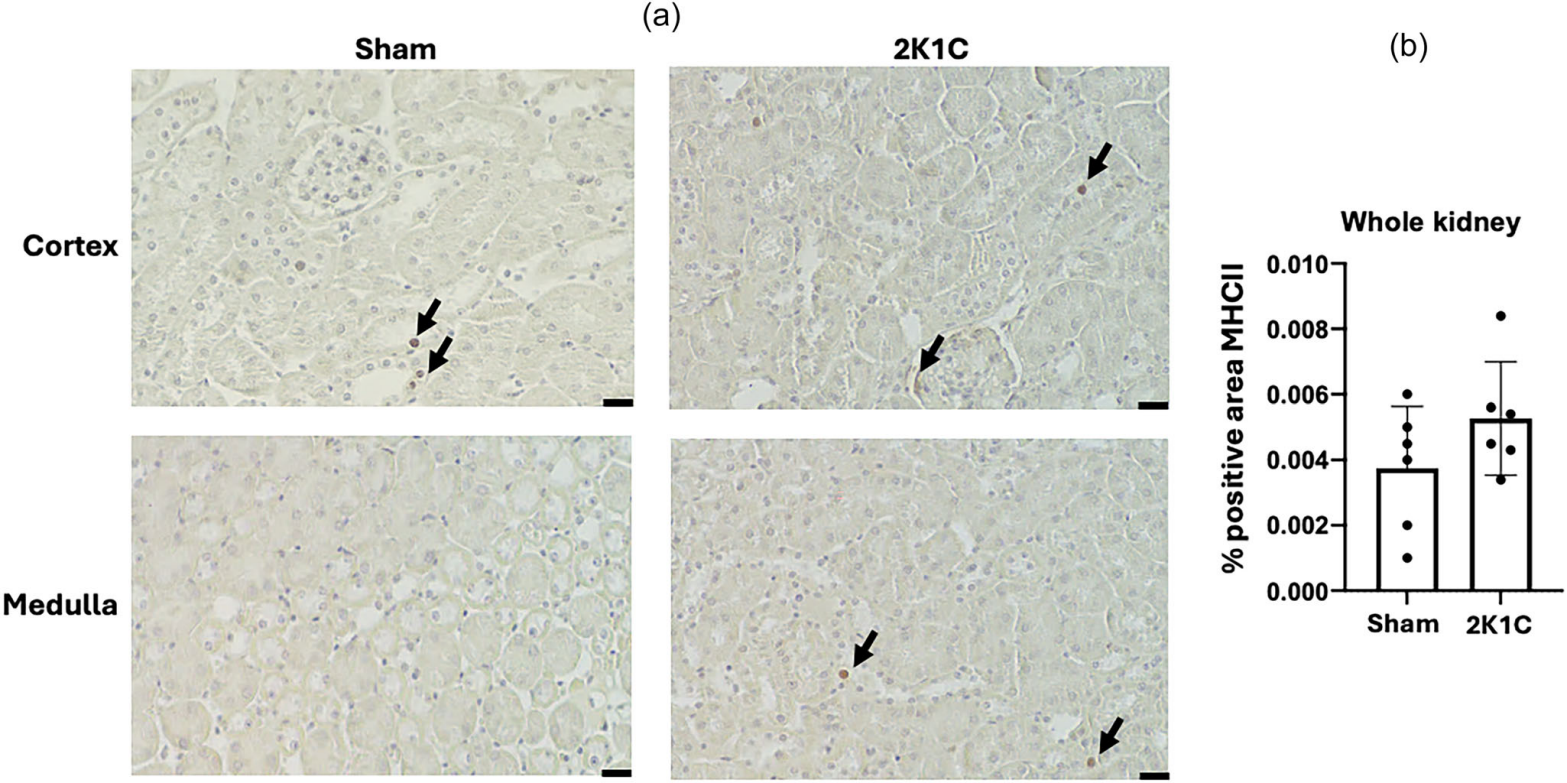
(a) Representative immunohistochemical staining for MHCII in renal cortex and medulla from Sham and 2K1C mice. MHCII‐positive staining was observed in kidneys from Sham and 2K1C mice in a conspicuous pattern likely in resident cells (arrows). (b) Quantification of whole‐kidney MHCII‐positive staining demonstrated no statistical differences between 2K1C mice compared with Sham controls. Data are presented as means ± SD. Scale bar: 20 µm.

Immunohistochemistry revealed MHC‐II^+^ cells distributed sparsely in the interstitium, peritubular areas and occasional glomerular regions in both Sham and 2K1C kidneys (Figure 5a). The pattern was at a very low density, consistent with resident APC populations. No qualitative increase in MHC‐II^+^ cell density or clustering was observed in 2K1C kidneys (Figure 5b).

## 4 DISCUSSION

The present study demonstrates that the early stage of renovascular hypertension induced by the 2K1C model is associated with the absence of structural renal remodelling and no global changes in the expression of APC, but focal increases of a marker of the monocyte/macrophage lineage F4/80. These findings support the concept that renovascular hypertension may not be exclusively related to haemodynamic changes, but rather a complex pathological condition which may promote early immune activation and tubular mechanisms that interact to sustain elevated blood pressure and progressive renal injury.

Our data confirm the activation of systemic RAS due to the increased JG renin expression (Figure 1b) in clipped kidneys, which was related to the increases in blood pressure (Figure 1c). These observations are consistent with the classical pathophysiological paradigm of renovascular hypertension, in which reduced renal perfusion distal to the arterial stenosis stimulates renin release from JG cells, promoting systemic Ang II generation and vasoconstriction (Prieto et al., 2021). We previously showed that the significant increases in systolic blood pressure observed at day 7 are accompanied by elevated intrarenal angiotensin‐converting enzyme (ACE) activity (Cardenas et al., 2024). These results reinforce the concept that the kidney possesses an autonomous intrarenal RAS capable of amplifying local Ang II formation independently of circulating peptide levels, which may have an impact on immune cell recruitment. Intrarenal Ang II is known to directly modulate tubular sodium transport, oxidative stress generation, inflammatory signalling and fibrotic pathways (Abadir et al., 2011; Cuevas, Gonzalez et al., 2015; Cuevas, Tapia‐Rojas et al., 2015; Mezzano et al., 2001; Siragy et al., 2003). Previous studies in experimental chronic kidney disease and obstructive nephropathy demonstrated that local renin and ACE expression are increased during renal injury, supporting the idea that local RAS activation participates in the progression of renal dysfunction independently of circulating renin levels (Figuero et al., 2019). Then, enhanced local Ang II generation within the renal parenchyma may contribute not only to vasoconstriction, but also to immune activation and sodium retention.

No significant increase in albuminuria was observed in 2K1C mice. Furthermore, no gross structural alterations in glomerular enlargement or expansion of Bowman's space were observed despite the expected Ang II‐mediated increases in glomerular capillary pressure. Since no changes in albuminuria were observed at day 7, and no other gross histological alterations were evident, it is possible that non‐haemodynamic mechanisms may contribute to renal injury during the establishment of hypertension (Araos et al., 2019; Satou et al., 2018; Shao et al., 2016). Inflammatory mediators, oxidative stress and immune cell infiltration are increasingly recognized as major contributors to glomerular permeability changes and podocyte dysfunction during hypertension.

Most of the evidence in the literature related to tissue injury in hypertensive models is described after 14 days. For instance, Ang II infusion models usually use 14 days of chronic infusions (Peng et al., 2017; Ramkumar et al., 2016; Wang et al., 2015). 2K1C models are usually studied after 14, 18 and 21 days after clipping (Prieto et al., 2003, 2011; Shao et al., 2016). Only a few studies have used this model during the first week (Lorenz et al., 2011). It has been shown that after 14 days of 2K1C, mice show that although both kidneys have elevated Ang II levels, the greater tissue injury is observed in the non‐clipped kidneys, indicating that an increased arterial pressure synergizes with increased intrarenal Ang II to exert greater renal injury (Shao et al., 2016).

Despite the absence of changes in protein levels of immune‐related markers MHC‐II and F4/80 expression within the kidney, the observation of the enhanced recruitment of macrophage lineage F4/80 during the first week of renovascular hypertension is particularly relevant considering the growing evidence implicating innate and adaptive immunity in the pathogenesis of hypertension (Araos et al., 2019). APCs, especially DCs and macrophages, are now recognized as active participants in blood pressure regulation and renal sodium handling rather than passive inflammatory players (Araos et al., 2019, 2025). Interestingly, MHCII immunostaining was evidenced in a few resident cells in both Sham and 2K1C kidneys, without differences in the number of positive cells.

Recent evidence demonstrated that renal APCs exposed to hypertensive stimuli acquire a prohypertensive phenotype capable of directly modulating renal sodium transport and promoting hypertension. In Ang II‐infused mice, renal APCs exhibit increased expression of oxidative stress‐related enzymes such as NADPH oxidase 2, inflammatory cytokines including IL‐1β, IL‐6, IL‐23, interferon‐γ and serum glucocorticoid‐regulated kinase 1 (SGK1), all of which are closely associated with sodium retention and immune activation. Moreover, adoptive transfer of renal APCs from hypertensive mice into normotensive recipient animals increases blood pressure and impairs natriuresis, establishing a causal relationship between renal APC activation and hypertension development (Araos et al., 2025).

The increased focal induction of F4/80 observed in our study strongly suggests an enhanced antigen presentation capacity within the kidney during the first stages of renovascular hypertension. This finding is highly relevant because APC‐mediated activation of T lymphocytes appears to be essential for the full hypertensive response in several experimental models. T cells contribute to hypertension by promoting cytokine production, vascular dysfunction, oxidative stress and modulation of tubular sodium transporters (Zhang et al., 2022). Experimental evidence demonstrates that mice deficient in T lymphocytes exhibit blunted hypertensive responses to Ang II and deoxycorticosterone acetate–salt treatment (Wenzel et al., 2019). Thus, the increase in renal APC markers identified in the present study likely reflects an active immune microenvironment capable of sustaining chronic inflammation and sodium retention.

The expansion of F4/80‐positive macrophages further reinforces the importance of innate immune activation during the early stages of renovascular hypertension. Macrophages contribute to renal injury through production of reactive oxygen species (ROS), cytokines and profibrotic mediators that amplify tubular dysfunction and interstitial remodelling and promote endothelial dysfunction, reducing nitric oxide bioavailability and increasing vascular resistance, worsening hypertension (Justin Rucker & Crowley, 2017). In addition, macrophages can influence renal tubular transport through paracrine signalling mechanisms that regulate sodium transporter activity (Ma et al., 2025). Therefore, the increase in macrophage infiltration observed in our study likely contributes not only to renal injury progression but also to altered sodium handling and maintenance of elevated blood pressure.

The interaction between the intrarenal RAS and the immune system appears to represent a critical feed‐forward mechanism during hypertension. Ang II promotes immune cell recruitment (Nataraj et al., 1999) and activation through stimulation of oxidative stress (Sedeek et al., 2009), cytokine production and endothelial adhesion molecule expression (Bovee et al., 2020). In turn, activated immune cells further enhance oxidative stress and inflammatory signalling, sustaining local RAS activation and sodium retention. This bidirectional crosstalk between immune pathways and the RAS may represent one of the central mechanisms underlying chronic hypertension and progressive renal injury. Furthermore, ROS generation can amplify intrarenal Ang II signalling and increase sodium transporter activity in distal nephron segments (Araos et al., 2025). These mechanisms collectively contribute to impaired natriuresis and maintenance of hypertension (Lins et al., 2021).

Although tubular transporters were not directly evaluated in the present study, our findings strongly suggest that altered tubular sodium handling is an important downstream consequence of immune activation and intrarenal RAS stimulation. Experimental studies demonstrated that immune cells regulate key sodium transporters including NKCC2, NCC, and ENaC (Norlander & Madhur, 2017). In Ang II‐dependent hypertension, APC depletion prevents NKCC2 activation and preserves natriuresis, supporting a direct role of immune cells in sodium transporter regulation (Araos et al., 2025). Therefore, it is plausible that the immune infiltration observed in our 2K1C model contributes to sodium retention through modulation of tubular transport pathways.

Another important aspect of our findings is the evidence supporting regional intrarenal heterogeneity in RAS and inflammatory responses. Previous studies demonstrated that cortical and medullary RAS components are differentially regulated during renal injury (Figuero et al., 2019; Nehme et al., 2019; Ramkumar & Kohan, 2016). In unilateral ureteral obstruction, cortical renin expression increases while medullary (pro)renin receptor expression decreases, suggesting compartment‐specific responses within the kidney (Figuero et al., 2019). These regional differences are highly relevant because cortical and medullary segments play distinct roles in sodium handling, oxygen consumption and inflammatory susceptibility. Although our study did not directly evaluate cortical versus medullary inflammatory responses, the global increase in immune markers likely reflects coordinated activation across multiple renal compartments.

Although we have explored the early stages of inflammatory events through the study of MHCII and F4/80 immune markers in the 2K1C renovascular model, our study has several limitations. First, functional characterization of infiltrating immune cells was not performed, limiting mechanistic conclusions regarding the specific immune cell subsets involved. Second, sodium transporter activity and tubular transport function were not directly assessed. Therefore, the relationship between immune infiltration and sodium handling remains inferential. Third, the cross‐sectional experimental design does not allow evaluation of temporal progression of immune activation and renal remodelling. Future studies should investigate whether immune cell infiltration precedes renal injury or develops secondarily to haemodynamic alterations. Additionally, characterization of cytokine profiles, oxidative stress pathways, and distal nephron sodium transporters would provide further mechanistic insight.

An interesting observation evidenced in Figure 5 is that MHC‐II immunostaining was considerably less abundant and exhibited a distinct spatial distribution compared with F4/80 staining, despite the well‐established capacity of renal macrophages to express MHC‐II. This apparent discrepancy may reflect the marked heterogeneity of renal resident macrophage populations. Recent studies have identified at least three major mononuclear phagocyte subsets in the healthy kidney, including resident macrophage subset 1 (F4/80 ‘high’, MHC‐II ‘low/intermediate’), resident macrophage subset 2 (F4/80 ‘high’, MHC‐II ‘high’), and monocyte/interstitial macrophages (F4/80 ‘intermediate’, MHC‐II ‘high’) (Cheung et al., 2022; Melkonian et al., 2025). Therefore, a substantial proportion of F4/80‐positive cells detected in the present study may correspond to resident macrophage populations expressing low levels of MHC‐II, which could explain why MHC‐II‐positive cells were less abundant than F4/80‐positive cells. In addition, MHC‐II expression is dynamically regulated throughout macrophage maturation, activation and differentiation, suggesting that macrophages present during this early stage of renovascular hypertension may represent different developmental or activation states (Wen et al., 2021). Consequently, the discordance between F4/80 and MHC‐II staining may reflect shifts in macrophage phenotype rather than differences in total mononuclear phagocyte abundance. Furthermore, because MHC‐II is also expressed by other professional APCs, including renal DCs, it is possible that a fraction of the MHC‐II‐positive cells identified in our study corresponds to non‐macrophage APC populations. Future studies combining additional markers and multiparametric approaches will be required to precisely define the identity and functional characteristics of these immune cell populations during the early phases of renovascular hypertension.

An important limitation of the present study is that the quantification of F4/80 immunostaining based on the percentage of positive area does not allow discrimination between an increased number of F4/80‐positive cells, enhanced F4/80 expression per individual cell, or changes in the spatial distribution of the immunoreactive signal. Consequently, this approach should be considered a semiquantitative measure of F4/80 immunoreactivity rather than a direct assessment of macrophage abundance. In this context, the increased F4/80‐positive area observed in the NCK is more appropriately interpreted as evidence of localized macrophage remodelling or activation, potentially reflecting the emergence of F4/80^hi^ microdomains, rather than a generalized increase in macrophage infiltration. This interpretation is consistent with the absence of significant changes in total F4/80 protein abundance detected by western blot, suggesting that early renovascular hypertension may induce focal alterations in macrophage phenotype or activation state before the development of widespread inflammatory cell accumulation. Future studies employing complementary approaches, including flow cytometry, multiplex immunofluorescence, or single‐cell transcriptomic analyses, will be required to distinguish between changes in macrophage number, phenotype and activation status and to define the functional significance of these localized immune microdomains during the early stages of renovascular hypertension.

Another limitation of our study is that all experiments were performed exclusively in male mice. This approach was adopted to minimize biological variability associated with the oestrous cycle and to maintain consistency with most previous studies using the 2K1C model. Nevertheless, biological sex is increasingly recognized as an important determinant of both the immune response and the pathophysiology of hypertension. Compared with males, premenopausal females generally exhibit a more anti‐inflammatory immune phenotype, characterized by greater regulatory T‐cell activity, enhanced IL‐10 production, and reduced renal infiltration of pro‐inflammatory T cells and macrophages, resulting in relative protection against hypertension and target‐organ damage (Comeau et al., 2022; Sylvester & Brooks, 2019). Conversely, males develop a more pronounced pro‐inflammatory response with increased Th17 polarization and greater renal immune cell infiltration (Comeau et al., 2022). Importantly, sex differences have also been reported in the 2K1C model, where female rodents exhibit an attenuated hypertensive phenotype, reduced renal injury and lower activation of the RAS, effects that are largely attributed to oestrogen‐mediated cardiovascular and immune protection (Lee et al., 2019). Therefore, future studies including female mice will be essential to determine whether the absence of early global immune activation observed in the NCK is conserved across both sexes and reflects sex‐dependent immunoregulatory mechanisms.

In summary, the present study demonstrates that early renovascular hypertension induced by the 2K1C model is characterized RAS activation, localized F4/80^+^ immune microdomain remodelling in the NCK, and the absence of overt structural renal remodelling. Rather than indicating a generalized immune cell influx, our findings support a model in which early renovascular hypertension promotes spatially restricted immune cell accumulation within discrete renal microdomains. This localized immune remodelling precedes overt structural injury and likely represents an early adaptive or pathogenic response to renovascular stress. Together, these findings refine our understanding of the early pathophysiology of renovascular hypertension and suggest that interactions between the intrarenal RAS and localized immune microenvironments may represent promising targets for interventions aimed at limiting disease progression before irreversible renal damage develops.

## AUTHOR CONTRIBUTIONS

Conceived and designed the study: Alexis A Gonzalez, Cristián A. Amador, and Carmen De Miguel. Performed experiments: Pilar Cárdenas, Allison C León, Catalina Cid‐Salinas, Juan Castillo‐Geraldo, Javiera Saez, Stefanny FIgueroa, Javier Reyes, Suarybell Guzmán Román, Emma Rosenkoetter. Analysed data: Pilar Cárdenas, Allison C León, Catalina Cid‐Salinas, Alexis A Gonzalez, Emma Rosenkoetter. Interpreted results of experiments: Pilar Cárdenas, Allison C León, Catalina Cid‐Salinas, Alexis A Gonzalez. Prepared figures: Pilar Cárdenas, Allison C León, Alexis A Gonzalez, Carmen De Miguel. Drafted manuscript: Pilar Cárdenas, Allison C León, Alexis A Gonzalez. Edited and revised manuscript: Pilar Cárdenas, Allison C León, Alexis A Gonzalez, Cristián A. Amador, Carmen De Miguel. All authors have read and approved the final version of this manuscript and agree to be accountable for all aspects of the work in ensuring that questions related to the accuracy or integrity of any part of the work are appropriately investigated and resolved. All persons designated as authors qualify for authorship, and all those who qualify for authorship are listed.

## CONFLICT OF INTEREST

None declared.

## GENERATIVE AI STATEMENT

The authors confirm that no artificial intelligence tools, including large language models (LLMs), were used in the drafting or revision of this manuscript. All content was conceived, written and approved solely by the authors.

## Data Availability

The source data are available to verified researchers upon request by contacting the corresponding author. All the supported data have been approved by the reviewers.
